# A Plea for Surgery in Pancreatic Metastases from Renal Cell Carcinoma: Indications and Outcome from a Multicenter Surgical Experience

**DOI:** 10.3390/jcm9103278

**Published:** 2020-10-13

**Authors:** Anna Caterina Milanetto, Luca Morelli, Gregorio Di Franco, Alina David, Donata Campra, Paolo De Paolis, Claudio Pasquali

**Affiliations:** 1 Clinica Chirurgica 1 - Pancreatic and Endocrine Digestive Surgical Unit. Department of Surgery, Oncology and Gastroenterology – University of Padua, via Giustiniani, 2 – 35128 Padova, Italy; davidalinagp@gmail.com (A.D.); claudio.pasquali@unipd.it (C.P.); 2 General Surgery Unit, Department of Translational Research and New Technologies in Medicine and Surgery, University of Pisa, via Paradisa, 2 – 56125 Pisa, Italy; luca.morelli@unipi.it (L.M.); gregorio.difranco@med.unipi.it (G.D.F.); 3 Chirurgia Generale e d’Urgenza 3, AOU Città della Salute e della Scienza di Torino, Corso Bramante, 88 – 10126 Torino, Italy; dcampra@cittadellasalute.to.it (D.C.); pdepaolis@cittadellasalute.to.it (P.D.P)

**Keywords:** renal cell carcinoma, pancreatic neoplasms, pancreatectomy, PET-CT scan

## Abstract

Background: Pancreatic metastases from renal-cell carcinoma (RCC-PMs) are rare. Surgery may play a role in improving overall (OS) and disease-free survival (DFS). Methods: Clinical-pathological features, surgery and follow-up data of patients with RCC-PMs operated on in three pancreatic surgical centers (2000–2019) were retrospectively evaluated. Results: Thirty-nine patients (21 male/18 female, averaging 65 years) were enrolled. RCC-PMs were metachronous in 36 patients (mean 94 months, up to 24 years after nephrectomy), multiple in 21 patients, and with a median size of 2.5 (range, 0.7–7.5) cm. All the patients underwent pancreatic surgery (33 standard resections, 6 limited resections). Fifteen patients had post-operative complications (morbidity 38.5%). The median DFS was 63 months, and 19 out of 36 patients showed a disease recurrence. The median OS was 134 months, and 13 out of 36 patients were alive with no evidence of disease. At univariate analysis, lymph node positivity (HR 5.1, 95% CI 1.5–18), multi-visceral resection (HR 3.4, 95% CI 1.1–10) and synchronous RCC-PMs (HR 13, 95% CI 3–55) were significantly associated with a short OS. Conclusion: Surgery may allow a DFS up to 17 years in more than one third of patients, even after limited resections. Splenectomy and lymph node dissection are not mandatory.

## 1. Introduction

Renal cell carcinoma (RCC) incidence rate has increased, and it accounts for 2%–3% of all new adult malignancies [1]. More than 50% of RCCs are currently detected incidentally [2], and about 20%–30% of patients have metastatic disease at presentation [3]. In localised RCC, 20%–30% of patients will have a recurrence after nephrectomy [4]; distant metastases from RCC occur mostly to the liver, lung and bone, and in 2.8% of patients may present in the pancreas [5]. Pancreatic metastases from RCC (RCC-PMs) are characterized by a slow growing behavior and a long interval of about 10 years before recurrence after nephrectomy [6]. 

Follow-up after nephrectomy which may include chest and abdominal imaging is rarely performed beyond five years, unless clinically indicated [7]. Therefore, RCC-PMs are often detected incidentally by abdominal ultrasound, computed tomography scan (CT) scan and/or magnetic resonance imaging (MRI) performed for other reasons in asymptomatic patients. Currently, a total body functional imaging is not part of the routine follow-up of these patients, although the systemic spread of RCC is well known. Both on CT scan and MRI, RCC-PMs show an early enhancement after contrast medium injection; MRI can detect RCC-PMs even without contrast-enhancement, as hyper intense lesions at T2- and diffusion-weighted images [8]. These imaging features are common to both RCC-PMs and pancreatic neuroendocrine neoplasms (pNENs), and the differential diagnosis with a non-functioning pNEN may be challenging [9]. So far, a previous nephrectomy was strongly suggestive of RCC-PM, but nowadays the endoscopic ultrasound-guided biopsy may help in differential diagnosis. 

Pancreatic surgery for RCC-PMs appears to confer a survival benefit to the patients [10], and a significantly longer overall survival (OS) when compared to PMs from other primary neoplasms may be achieved (median 109 vs. 36 months, respectively) [11]. A surgical treatment may have a role in improving OS and disease-free survival (DFS) in patients with RCC-PMs, even in the era of anti-vascular endothelial growth factor agents. Since the treatment with tyrosine kinase inhibitors (TKIs) failed to show a complete objective response in patients with metastatic setting [6], the question is whether surgery may be extended to a larger number of patients to give them OS and/or DFS benefits. This retrospective study collects the experience of three Italian high-volume pancreatic surgical centers on RCC-PM in the last 20 years. Clinical-pathological features, surgical management and DFS/OS were evaluated, and indications to pancreatic surgery and follow-up timing and imaging were discussed. 

## 2. Patients and Methods

Clinical records of patients with RCC-PMs observed from November 2000–December 2019 in three Italian high-volume pancreatic surgical centers (Surgery Unit 1, Padua; General Surgery Unit, Pisa; and General and Emergency Surgery, Turin) were retrieved retrospectively. Patients who underwent surgery for a RCC-PM in the study period were enrolled. The following data were analyzed: age (years), gender, date and type of surgery for RCC; RCC staging (according to AJCC classification 8th ed.) [12]; date of diagnosis of PM (with “synchronous RCC-PM” defined as a PM diagnosed at the same time, or within six months of the RCC diagnosis); disease-free interval (DFI, defined as the time from resection of the primary RCC to the onset of PM); cross-sectional imaging studies (CT scan, MRI); functional imaging studies, such as ^18^F- fluorodeoxyglucose positron emission tomography-CT (^18^F-FDG PET-CT), ^68^Gallium-DOTA-peptide PET-CT (^68^Ga PET-CT), and ^111^In-Octreotide scintigraphy; number and pancreatic location of PM; extra-pancreatic sites of RCC metastases. 

We considered type of pancreatic surgery (standard resections: pancreatico-duodenectomy, distal pancreatectomy, total pancreatectomy; and limited resections: spleen-preserving distal pancreatectomy, duodenum-preserving pancreatic head resection, central pancreatectomy, enucleation); associated abdominal surgery (including splenectomy, and multi-visceral resections); operative time (min), blood loss (mL), and hospital stay (days). Surgical outcome included overall morbidity, early post-operative mortality (within 30 days from surgery), post-pancreatectomy haemorrhage, delayed gastric emptying, and post-operative pancreatic fistula (according to the International Study Group on Pancreatic Fistula definition) [13]. The following histological data were evaluated: tumor size (cm), lymph node metastases and lymph node ratio (defined as the number of positive lymph nodes on the total number of lymph nodes analyzed). 

Follow-up closed at 31st December 2019. In all the patients with at least six months of follow-up we evaluated OS and DFS, defined by using a personal telephone interview or at the last follow-up visit, that included clinical evaluation and imaging studies (CT scan, MRI and/or ^68^Ga PET-CT) to detect any tumor recurrences. 

Kaplan-Meier survival curves for OS and DFS were plotted and compared using the log-rank test. The Chi-square test or Fisher’s exact test were used for comparison of categorical variables, when appropriate. Cox proportional-hazard models were used to identify risk factors associated with OS and DFS at univariate analysis. The results were reported as hazard ratios (HRs) and 95% confidence intervals (95% CI). A p value less than 0.05 was considered statistically significant. Statistical analysis was performed using the R program version 3.6.3 [14].

### Statement of Ethics

The research was conducted ethically in accordance with the World Medical Association Declaration of Helsinki. Subjects gave their written informed consent to data processing anonymously for research purposes. The ethics committee of the Azienda Ospedaliera di Padova approved the present study (project code: 2872p). 

## 3. Results

Thirty-nine patients with RCC-PMs underwent open pancreatic resection and were enrolled in the study. Clinical features, surgery, and post-operative outcome are described in Table 1. 

**Figure 1:**
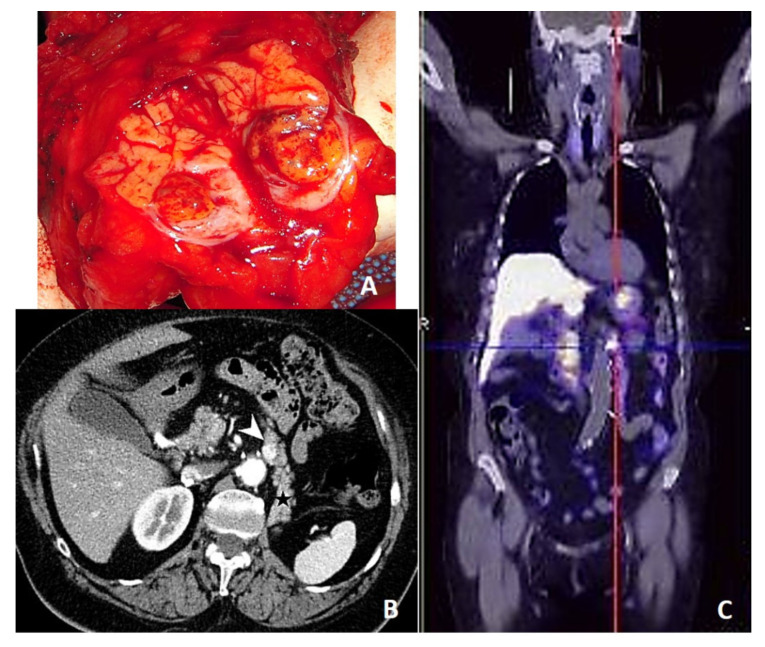
(**A**) Macroscopic appearance of a RCC-PM after cutting the specimen; (**B**) preoperative CT scan (white arrow for the lesion, black star for the pancreatic tail); (**C**) ^68^Ga PET-CT of a single 1.2 cm-in size RCC metastasis in the pancreatic body.

There were 21 men and 18 women, averaging 65 years (range, 45–81 years). All primary RCC were treated by R0 radical nephrectomy; all the patients had a clear cell RCC, and only one patient had histologically confirmed lymph node metastases. In 36 patients RCC-PMs were metachronous lesions, occurring after a median time of 84 (range, 7–291) months, and in one third of patients after a DFI longer than 10 years. In 21 patients RCC-PMs were multiple lesions, located in the body-tail of the pancreas in 28 cases (despite a left-sided nephrectomy in 23 patients), and with a median size of 2.5 (range, 0.7–7.5) cm. Only seven patients showed concomitant extra-pancreatic metastases. Concerning functional imaging studies, ^18^F-FDG PET-CT showed a moderate tracer uptake in 9 out of 14 patients; ^68^Ga PET-CT demonstrated a strong tracer uptake in all three patients in which it was performed (Figure 1), and when considered together, ^68^Ga PET-CT and ^111^In-Scintigraphy were strongly positive in five out of seven patients. 

All the patients underwent open pancreatic surgery with curative intent, and in five patients a multi-visceral resection was needed due to other abdominal metastases. A pre-operative diagnosis was correctly assessed in 30 patients by imaging studies and medical history. Surgery consisted in 33 standard pancreatic resections (13 distal pancreatectomy, 12 total pancreatectomy, and eight pancreatico-duodenectomy), and six limited resections (three spleen-preserving distal pancreatectomy, one duodenum-preserving pancreatic head resection, and two central pancreatectomy). Ten patients underwent multi-visceral resections, which included: nephrectomy/ipsilateral adrenalectomy (*n* = 2) and hemicolectomy/ureteral resection (*n* = 1) for a synchronous RCC, partial/total gastrectomy (*n* = 3), hemicolectomy right/left (*n* = 2), adrenalectomy ipsilateral/contralateral (*n* = 3), and partial vena cava resection (*n* = 1). Histology showed an average of 18 lymph nodes counted, with an involvement of peri-pancreatic lymph nodes in five patients. Additional splenectomy was performed in 22 patients, and no splenic secondary lesions were detected. Fifteen patients had post-operative complications (morbidity 38.5%). Notably, there were four post-operative pancreatic fistulas grade B, and two post-pancreatectomy haemorrhages (in the standard resection group). Other post-operative complications included: pneumonia/pulmonary atelectasis (*n* = 6), respiratory failure (*n* = 1), splenic infarction after Warshaw operation (*n* = 1), and renal failure (*n* = 1). One patient with a liver hematoma required a reoperation, and one patient died from pulmonary embolism (mortality 2.6%). 

Thirty-six patients were considered for the analysis of long-term outcome and survival as DFS and OS. Long-term follow-up is described in Table 2.

**Figure 2:**
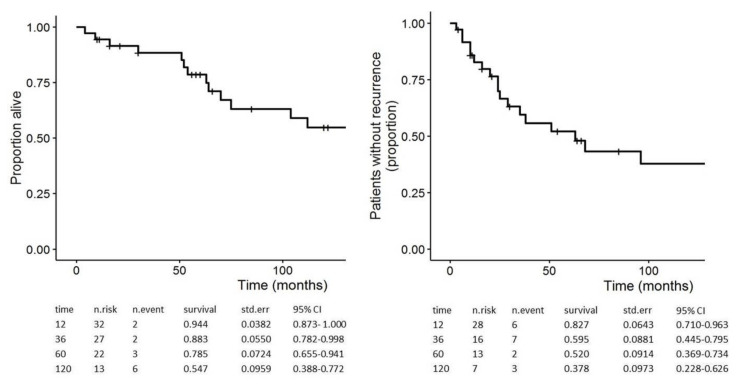
Kaplan-Meier estimates for overall survival (OS) (left) and disease-free survival (DFS) (right) of 36 patients after pancreatic surgery for RCC-PM.

After a median follow-up of 68 (range, 4–201) months, post-operative diabetes and exocrine insufficiency were observed in 17 out of 36 and in 15 out of 35 patients, respectively. In the limited resection group, all the patients had normal endocrine and exocrine pancreatic functions. After a median DFS of 63 (range, 3–201) months, 19 patients experienced a disease recurrence, located in the pancreas in five cases. Notably, 4 out of 19 patients in follow-up after standard resection (excluding total pancreatectomy) showed a pancreatic recurrence after a median DFS of 20 months. When considering the 31 patients in follow-up with PMs only, 16 of them showed a recurrence after a median DFS of 25 (range, 3–130) months, and their median residual survival after recurrence was 50 (range, 4–121) months. The 1-, 3-, 5-, and 10-year DFS rates were 83%, 60%, 52%, and 38%, respectively (Figure 2). Finally, 11 patients in follow-up (median 68 months) died of disease; 20 patients were still alive, and 13 of them without evidence of disease. The median OS was 134 months; the 1-, 3-, 5-, and 10-year OS rates were 94%, 88%, 79%, and 55%, respectively (Figure 2). 

At Cox-regression univariate analysis (Table 3), lymph node positivity (HR 5.1, 95% CI 1.5–18), multi-visceral resection (HR 3.4, 95% CI 1.1–10) and synchronous RCC-PMs (HR 13, 95% CI 3–55) were significantly associated with a short OS. 

## 4. Discussion

Nephron-sparing or radical nephrectomy is the standard of care for localized RCC, with a 20-30% of recurrence rate [15], and RCC-PMs are reported to occur in 2.8% of patients [5]. In our experience, RCC-PMs were located in the body-tail of the pancreas in 28 cases (irrespective of the side of nephrectomy), multiple in 21 patients, and only seven patients had extra-pancreatic metastases. Confirming previous reports [6,16], 92% of RCC-PMs arose as metachronous, occurring after a median DFI of seven years (up to 24 years) after nephrectomy. Occasionally, metastases may have occurred earlier and been overlooked by the cross-sectional imaging studies [9]. The gold standard to detect hyper vascular RCC-PMs in the follow-up of RCC patients should be the CT scan, and MRI could be an acceptable alternative option [8]. One third of our patients developed RCC-PMs after a DFI of more than 10 years; a long follow-up over 10 years should be considered, even in asymptomatic patients. Due to the systemic spread of RCC, functional total-body imaging studies may have some relevance in the follow-up after nephrectomy for RCC. In our series, ^18^F-FDG PET-CT had a sensitivity of 64% in detecting RCC metastases, as previously reported [17]. However, RCC-PMs showed a low/moderate FDG uptake, whereas ^68^Ga PET-CT and ^111^In-Scintigraphy together (both binding to somatostatin receptors) showed a strong positivity in five out of seven patients. The positivity of ^111^In-Scintigraphy is a common finding in metastatic RCC [18]. Hence, ^68^Ga PET-CT (which replaced ^111^In-Scintigraphy) could be a promising tool for the RCC staging, and it may be advisable once RCC recurrence or metastases are suspected at CT scan. 

In metastatic setting of RCC, the resection of RCC metastases is recommended as local treatment for most sites, except brain and bone metastases [19], since the surgical treatment can lead to a long DFS, even without a systemic therapy [20]. Concerning RCC-PMs, five-year survival was significantly longer in patients who underwent surgery (72–73%) when compared to patients without surgery (0–14%) [10,16]. In a recent multicenter study [6], surgery and TKIs showed comparable results, although evaluated as PFS and DFS, respectively, but patients treated with TKIs had no complete responses [6]. Moreover, TKI-related toxicity has been observed in most patients, with a decline in quality of life [21]. In our surgical series, we obtained a complete objective response in 13 patients, in terms of patients living without disease after a median follow-up of 148 months. In our study, in RCC-PMs without extra-pancreatic metastases, 19 showed a recurrence after a median DFS of 25 months, and their median residual survival after recurrence was 50 months. Even in a subset of patients older than 65 years, and those with a stage III-IV RCC at diagnosis, the five-year OS was found to be 80% and 71%, respectively, after pancreatic surgery. In our study, mortality rate was 2.6%, clinically relevant post-operative pancreatic fistula (excluding total pancreatectomy) occurred only in 15% of cases, and 62% of patients had an uneventful post-operative course. These results are in line with a recent study on “high-risk complication” patients who underwent pancreatic surgery [22], with morbidity and mortality rates of 42.5% and 3.5%, respectively. Particularly, morbidity and mortality rates of patients who underwent major pancreatic surgery (total pancreatectomy and pancreatico-duodenectomy) was 40% and nil, respectively. Thus, when performed in centers specialized in pancreatic surgery, a correct management of expected complications will minimize the risk of severe outcomes, and an aggressive approach for the treatment of RCC-PMs can be adopted to pursue the goal of an R0 resection, even if this could require a major pancreatic resection. 

In the univariate analysis we analyzed the possible impact of the presence of single/multiple RCC-PMs, of the presence of extra-pancreatic disease, and of the DFI on the DFS and OS. High-volume analyses during the last 10 years on RCC-PMs showed that results of RCC-PMs surgical resection are not conditioned by the presence of single or multiple metastases, or the presence of synchronous or metachronous metastases [23,24,25,26]. In our study we also reported no difference in DFS or OS at Cox-regression univariate analysis between single or multiple metastases. On the contrary, at univariate analysis a significantly (*p* < 0.001) shorter OS was associated with synchronous RCC-PMs when compared to metachronous lesions with a HR of 13 (95% CI 3–55). However, this may be the result of low statistical power due to the small sample size of the patients with synchronous RCC-PMs. Furthermore, RCC-PMs without extra-pancreatic disease carry a favorable prognosis with a cumulative five-year survival after surgery of up to 88% [27], even if this finding was not confirmed in our study. A prolonged DFI is a characteristic feature of patients with RCC pancreatic metastases, and in our series, we reported 13 patients with a DFI of more than 10 years, nine of which were still alive without evidence of disease. Nevertheless, no associations between DFI and survival were observed, even when raising the DFI cut-off from 5 to 10 years, in accordance with data published recently in the literature [24,26,28]. Therefore, when a radical resection of RCC-PMs is obtainable, this should be taken in consideration both in the case of single or multiple RCC-PMs, or extra-pancreatic disease, and independently by the DFI. Moreover, we observed a disease recurrence in about half of patients either with single or multiple PMs, confirming a previous study [16]. However, as reported by Di Franco at al., an aggressive treatment could be taken into consideration also in case of recurrent disease after RCC pancreatic metastases resection and multiple surgical treatment of recurrent RCC metastases, diagnosed during the follow-up [11].

The usefulness of lymphadenectomy in pancreatic surgery performed for RCC-PMs is still debated. Several previous reports found that a peri-pancreatic lymph node involvement with PMs is uncommon [29], or even absent [30], especially with the pancreas as the only metastatic site [16]. In our study, only five patients had a RCC involvement of peri-pancreatic lymph nodes, but they showed a worse five-year OS than patients with negative lymph nodes (53 vs. 82%, respectively), confirmed at univariate analysis (*p* = 0.011). From our experience, lymph-node dissection should be performed only in case of suspected lymph node involvement detected intra- or pre-operatively. Similarly, splenectomy could be performed only for a pancreatic tail lesion close to the hilum, or if a lymph node involvement at the splenic hilum is suspected, since splenectomy was not related to any survival advantages. Some authors reported a high local recurrence rate after limited resections for RCC-PMs [31], but in our study no significant differences were shown between standard and limited resections in terms of DFS/OS. Considering long-life expectancy of these patients, limited pancreatic resections may be an alternative to standard procedures. Pancreatic recurrence occurred in five out of 24 patients in follow-up after pancreatic surgery (excluding total pancreatectomy), and all but one patient had undergone a standard pancreatic resection. Thus, a pancreatic “recurrence” may be related to undetected multiple PMs [32], rather than to the surgical procedure. Moreover, disease relapse after pancreatic surgery occurred mostly in distant extra-pancreatic sites. From our experience, a standard pancreatic resection with lymph node dissection (and splenectomy) may not prevent pancreatic (and systemic) recurrence of disease, since DFS depends mostly on extra-pancreatic metastases. Multi-visceral resections, performed with curative intent and mostly associated with total pancreatectomy, showed a significantly short OS (*p* = 0.029), confirming the bad prognosis when infiltration includes surrounding organs. Moreover, total pancreatectomy results in exocrine insufficiency and insulin-dependent diabetes mellitus, which may affect the global quality of life, especially for relatively young patients who had undergone nephrectomy. 

Our study has some limitations due to the relatively small number of patients included and the retrospective design and data collection. Moreover, similarly to other published studies, the lack of a control group represents another limitation. In fact, the multicenter nature and the long period of the study made it difficult to obtain a really comparable control group of patients that did not undergo surgical resection in the last 20 years. However, patients with pancreatic metastases treated with sole systemic therapies were either patients not fit for surgery because of severe comorbidities with a high operative risk for pancreatic surgery, or patients not susceptible to undergo a R0 resection because of plurimetastatic disease with diffuse extra-pancreatic localizations. Therefore, any comparison with such group could have introduced a selection bias, because patients who were not operated on were more likely to have shorter survival rates. Nevertheless, this study reported the experience of three tertiary referral centers for pancreatic surgery, with a long time of follow-up. 

In conclusion, the assessment of RCC-PMs may be improved using functional total body imaging, particularly ^68^Ga PET-CT as a second-line imaging technique, and follow-up after nephrectomy for RCC should be extended after 10 years. Indications to surgery should be taken in consideration for RCC-PMs in which a radical resection could be obtained, both for single and multiple PMs; we have insufficient data to extend indications to patients with extra-pancreatic disease. Limited pancreatic resections are equivalent in terms of recurrence to standard pancreatic procedures, and splenectomy and lymph node dissection are not mandatory, since lymph node involvement is uncommon. In pancreatic units, resective surgery may obtain a complete objective response, with more than one third of patients living without disease after a median follow-up longer than 12 years. Moreover, in our opinion, with the introduction of new possible locally directed therapy, such as surgical, ablative or radiation-based approaches, and of new chemotherapy drugs, today each case of RCC-PM should be treated thorough multidisciplinary evaluation performed by general surgeons, radiation therapists, interventional radiologists, and medical oncologists. Probably, combination therapy with the newly available target therapies will be crucial for the management of these patients in the future. 

## Figures and Tables

**Table 1:** Clinical features, surgery, and post-operative outcome (*n* = 39).

Parameter	
Age (years), median (range)	65 (45–81)
Sex, *n*	
Male	21
Female	18
Nephrectomy, *n*	
Left	23
Right	16
Stage RCC [12], *n*	
I	2
II	14
III	20
IV	3
Timing of RCC-PM, *n*	
Synchronous	3
Metachronous	36
Disease free interval (months), median (range)	84 (7–291)
Number of RCC-PM, *n*	
Single	18
Multiple	21
Other sites of metastases, *n*	
No	32
Abdominal	3 local relapse, 1 contralateral kidney, 1 adrenal
Extra-abdominal	1 lung, 1 thyroid
Pancreatic metastases size (cm), median (range)	2.5 (0.7–7.5)
Type of pancreatic surgery, *n*	
Distal pancreatectomy	13
Total pancreatectomy	12
Pancreatico-duodenectomy	8 (1 associated enucleation)
Limited resections	3 spleen-preserving DP, 1 DPPHR, 2 CP (1 associated enucleation)
Associated surgery, *n*	
Splenectomy	22
Multi-visceral resections	12
Operation time (min), median (range)	350 (150–720)
Intra-operative blood loss (mL), median (range)	400 (100–1300)
Post-operative morbidity*, n*	
Post-operative pancreatic fistula	9 (5 biochemical leak, 4 post-operative pancreatic fistula grade B)
Post-pancreatectomy hemorrhage	2
Delayed gastric emptying	1
Other cause	9
Post-operative mortality, *n*	1
Reoperation rate, *n*	1
Hospital stay (days), median (range)	12 (6–133)

CP–central pancreatectomy, DP–distal pancreatectomy, DPPHR–duodenum-preserving pancreatic head resection, n.a.–not available, RCC renal cell carcinoma, RCC-PM–pancreatic metastasis from renal cell carcinoma.

**Table 2:** Long-term follow-up (*n* = 36).

Parameters	
Post-operative diabetes, *n*	17
Post-operative exocrine insufficiency, *n*	15
Disease recurrence, *n*	19
Site of recurrence, *n*	
Liver	7
Lung	7
Pancreas	5
Bone	2
Thyroid	1
Adrenal	1
Follow-up (months), median (range)	68 (4–201)
Status, *n*	
Died of disease	11
Died of other cause	5
Alive with disease	7
Alive and no evidence of disease	13

**Table 3:** Univariate Cox regression analysis for OS and DFS (*n* = 36).

	OS				DFS			
	Event	HR	95% CI	*p* Value	Event	HR	95% CI	*p* Value
Age (years)	16				19			
<65 (*n* = 17)	9	1			11	1		
≥65 (*n* = 19)	7	0.97	0.36–2.6	0.95	8	0.84	0.34–2.1	0.70
Stage RCC	16				19			
I-II (*n* = 13)	5	1			8	1		
III-IV (*n* = 23)	11	2.9	0.89–9.4	0.077	11	0.93	0.37–2.3	0.88
Timing RCC-PM	16				19			
Metachronous (*n* = 33)	13	1			17	1		
Synchronous (*n* = 3)	3	13	3–55	<0.001	2	4.2	0.91–19	0.065
Number RCC-PM	16				19			
Single (*n* = 16)	6	1			9	1		
Multiple (*n* = 20)	10	1.1	0.38–3	0.9	10	0.79	0.32–1.9	0.61
Extra-pancreatic metastases	16				19			
No (*n* = 30)	14	1			16	1		
Yes (*n* = 6)	2	2.8	0.54–15	0.22	3	2.0	0.57–7.4	0.27
Pancreatic resection	16				19			
Limited (*n* = 5)	2	1			4	1		
Standard (*n* = 31)	14	1.8	0.41–8.3	0.43	15	0.58	0.19–1.8	0.34
Multi-visceral resection	16				19			
No (*n* =26)	10	1			13	1		
Yes (*n* = 10)	6	3.4	1.1–10	0.029	6	1.8	0.66–4.7	0.25
Splenectomy	16				19			
No (*n* =15)	8	1			10	1		
Yes (*n* = 21)	8	0.94	0.35–2.5	0.89	9	0.74	0.3–1.8	0.52
Postoperative complications	16				19			
No (*n* = 23)	10	1			9	1		
Yes (*n* = 13)	6	0.84	0.3–2.3	0.74	10	1.9	0.77–4.7	0.17
Size RCC-PM (cm)	16				19			
<2.5 (*n* = 14)	10	1			6	1		
≥2.5 (*n* = 22)	6	1.1	0.4–3.2	0.80	13	1.7	0.63–4.5	0.31
Lymph-node positivity	16				19			
No (*n* = 31)	12	1			16	1		
Yes (*n* = 5)	4	5.1	1.5–18	0.011	3	1.4	0.4–5.1	0.59
Recurrence	16							
No (*n* = 17)	4	1			-	-	-	
Yes (*n* = 19)	12	2.6	0.81–8.1	0.11	-	-	-	-
DFI (months)	13				17			
≥60 (*n* = 22)	9	1			12	1		
<60 (*n* = 11)	4	1	0.31–3.4	0.96	5	0.96	0.34–2.7	0.94

DFI–disease-free interval; DFS–disease-free survival; OS–overall survival; RCC–renal cell carcinoma; RCC-PM–pancreatic metastasis from renal cell carcinoma.
